# Association between lipid metabolism and periodontitis in obese patients: a cross-sectional study

**DOI:** 10.1186/s12902-023-01366-7

**Published:** 2023-05-25

**Authors:** Ru Jia, Yuwei Zhang, Zhiyu Wang, Bo Hu, Zhenzhen Wang, Hu Qiao

**Affiliations:** 1 Key Laboratory of Shaanxi Province for Craniofacial Precision Medicine Research, Clinical Research Center of Shaanxi Province for Dental and Maxillofacial Diseases, College of Stomatology, Xi’an Jiaotong University, No. 98 Xiwu Road, Xi’an, Shaanxi 710004 China; 2 Department of Prosthodontics, College of Stomatology, Xi’an Jiaotong University, Xi’an, Shaanxi China; 3 Department of Stomatology, Hospital of Xi’an Jiaotong University, Xi’an, Shaanxi China; 4 Department of Orthodontics, College of Stomatology, Xi’an Jiaotong University, Xi’an, Shaanxi China

**Keywords:** Obesity, Periodontitis, Lipid metabolism, Inflammatory cytokines, Adipokines

## Abstract

**Background:**

To investigate the association between clinical periodontal parameters of periodontitis, serum lipid metabolism markers and adipokines’ levels in patients with obesity and periodontitis.

**Methods:**

A total of 112 patients admitted to Hospital of Xi’an Jiaotong University were included in this study. They were divided into normal body weight group (18.5 < body mass index, BMI < 25, n = 36), overweight group (25 ≤ BMI < 30, n = 38), and obesity group (BMI ≥ 30, n = 38) accordingly. The diagnosis of periodontitis was based on the newest international classification of periodontitis. Full-mouth clinical periodontal measurements included: plaque index, periodontal pocket depth, clinical attachment level, and bleeding on probing. Gingival crevicular fluid samples were analyzed for: Interleukin-1β, tumor necrosis factor-α, Interleukin-6 and C-reactive protein. Serum triglycerides, total cholesterol, low density lipoprotein cholesterol, high density lipoprotein cholesterol and glycosylated hemoglobin levels were measured. Visfatin, leptin, resistin, and adiponectin levels in serum were also measured.

**Results:**

The ratio of participants without periodontitis was significantly highest in normal weight group, and the proportion of severe periodontitis (stage III and IV) was highest in obesity group. The periodontal pocket depth, clinical attachment level, and the inflammatory cytokines in gingival crevicular fluid in obesity group and overweight group were higher than those in normal body weight group. The BMI and waist-to-hip ratio (WHR) were significantly positive correlated with periodontal pocket depth and clinical attachment level. Using a Multivariate logistic regression model, periodontitis correlates to BMI, WHR, serum levels of triglyceride, total cholesterol, low density lipoprotein, and adipokines such as visfatin, leptin, and resistin.

**Conclusions:**

Obesity is positively correlated with the aggravation of periodontitis. Obesity may aggravate the damage to periodontal tissue by regulating the secretion level of adipokines.

## Introduction

Periodontitis is a chronic inflammatory and multifactorial disease of periodontal supporting tissue initiated by bacteria [1, 2]. Periodontitis usually develops slowly and chronically, presenting symptoms that may not be easily noticed, and often leads to neglect and loss of treatment opportunities, which usually become the main cause of advanced alveolar bone destruction and tooth loss in adults. This not only reduces chewing function but also affects the speaking and aesthetics of the patients. Moreover, it may result in systemic complications, such as pneumonia, chronic obstructive pulmonary disease, diabetes, and cardiovascular diseases, affecting the patient’s quality of life seriously [3–5].

In recent years, a large number of systematic studies have shown that there is a strong epidemiological relationship between periodontitis and obesity [6]. Obesity is an increasingly prevalent worldwide disease with a high prevalence rate in both developed and developing countries. Approximately 56% and 78% of the world’s female and male populations, respectively, are obese [7]. A large number of epidemiological studies have shown that compared to people with normal body weight, overweight/obese adults and adolescents have a higher susceptibility to periodontitis under the same influence of local stimulus factors [8]. Body weight, body mass index (BMI) and waist-to-hip ratio (WHR) were positively correlated with periodontal pocket depth and attachment loss in overweight/obese patients [9]. Animal experimental studies have shown that the ligation-induced local periodontal inflammation was more severe and alveolar bone loss was more obvious in obese animals [10]. More importantly, periodontitis patients with obesity have significantly worse results after periodontal non-surgical treatment than those with normal weight [11]. Although previous studies have suggested that obesity may promote the occurrence of periodontitis [12–17], few studies shed light on the nature and the underlying mechanism of the relationship between these two diseases [18].

As we know, obesity is characterized by excessive accumulation of adipose tissue. The abnormal secretion function of adipose tissue caused by excessive accumulation (decreased secretion of anti-inflammatory adipokines and increased secretion of pro-inflammatory adipokines) is the inducement of systemic chronic inflammatory response [18, 19]. Adipokines act on the numerous organs of the body through various secretory pathways and play a vital role in regulating food intake, energy metabolism, and inflammatory response, etc. [20]. As abnormal expressions of adipokines such as visfatin [21], adiponectin [22] and leptin [23] have been found in the periodontal tissues of patients with periodontitis, the important role of adipokines in periodontitis has attracted more and more attention from researchers. Therefore, the purpose of this study is to investigate the association between clinical periodontitis measures with serum lipid metabolism indicators and adipokine levels in normal weight, overweight and obese people. The present study is also meant to present biological mechanisms considering obesity as a risk factor for periodontitis and provides a more theoretical basis for the prevention and treatment of periodontitis in obese patients.

## Materials and methods

### Participants

112 patients admitted to Hospital of Xi’an Jiaotong University for oral examination from July to December 2020 were recruited for this cross-sectional study in total. BMI and WHR of each patient were recorded and calculated as indicators of obesity according to the classification standards of obesity degree of the World Health Organization (BMI calculated as kg/m^2^, 18.5 < BMI < 25 was considered normal, 25 ≤ BMI < 30 was considered overweight, and BMI ≥ 30was considered obese). There were 36 normal weight patients (18 males, age 39 ± 1.5 years, 18 females, age 40 ± 2.7 years), 38 overweight patients (21 males, age 43 ± 2.5 years, 17 females, age 41 ± 1.9 years), and 38 obese patients (20 males, age 42 ± 2.1 years, 18 females, age 39 ± 3.1 years). Exclusion criteria for any of the known effects of periodontal health status, such as pregnant and lactating women, diabetics, orthodontic treatment, periodontal treatment within the past 6 months, lipid-lowering therapy, using immunosuppressant, cnon-steroid anti-inflammatory drugs and antibiotics within the past 3 months, and less than 10 teeth remaining in the mouth. Heavy smokers (>20 cigarettes/day) were also excluded in this study. This study was approved by the Biomedical Ethics Committee of Xi ‘an Jiaotong University Medical School (Approval No. 2020 − 385), and it was certified that the study was performed in accordance with the 1964 declaration of Helsinki and later amendments. The written informed consent of all the participants were obtained prior to clinical periodontal examination and intravenous blood collection.

### Clinical periodontal measurements

According to the periodontitis staging criteria in the current international classification of periodontitis established in the 2017 International Symposium on the Classification of Periodontitis and Perimplant Diseases [24], the participants underwent a professional oral examination. Full-mouth clinical periodontal measurements of all teeth (except for the third molars) were recorded at six sites (mesio-buccal, buccal, disto-buccal, disto-lingual, lingual and mesio-lingual), including visible periodontal plaque index (%), periodontal pocket depth (mm), clinical attachment level (mm), and bleeding upon probing (Yes/No) using a periodontal probe. All periodontal examinations were performed by the same periodontist, who had been previously trained and was unaware of the case-control status. Each measurement was performed three times and the average value was recorded and collected finally.

### Serum and gingival crevicular fluid (GCF) samples

After 12 h of fasting, 2 ml of the venous blood samples from the patients were taken and placed in a vacuum tube, then centrifuged at 1500 rpm/min for 10 min for biochemical screening tests. The serum was separated and stored at -20 °C until examination.

GCF was sampled from the sites with the deepest periodontal pocket depth by placing the paper strips gently at the entrance of the gingival sulcus for 30 s, and then stored in dry microcentrifuge tubes. The microcentrifuge tubes were weighed. The concentration of 0.15 mg:150µL was set as 1, and the corresponding ratio of 150 µl ELISA kit with buffer solution for dilution was added. The solution was centrifuged at 1500 rpm/min at 4 °C for 15 min, and 100 µl supernatant was extracted and stored in a refrigerator at -80 °C for later use.

### Biochemical analysis

The biochemical analysis was performed on all 112 samples. The samples of serum and GCF to be tested were balanced at room temperature for 20 min, centrifuged at 3 000 rpm/min at 4 °C for 10 min. Blood glucose was determined using OneTouch SureStep Test Strips (Johnson &Johnson, New Brunswick, NJ, USA). Serum triglycerides, total cholesterol, low density lipoprotein cholesterol (LDL-C), high density lipoprotein cholesterol (HDL-C) and glycosylated hemoglobin (HbA1c) levels were measured using commercially assays kits (Beijing Puerweiye BioTech, Beijing, China). Concentrations of adipokines including visfatin, leptin, resistin, and adiponectin and concentrations of inflammatory cytokines including C-reactive protein (CRP), Interleukin-6 (IL-6), Interleukin (IL-1β), and tumor necrosis factor-α (TNF-α) in serum and GCF were measured using the corresponding ELISA kit (Thermo scientific, Waltham, MA, USA) respectively on a Microplate Absorbance Reader (Bio-Rad Laboratories, Hercules, CA, USA).

### Statistical analysis

The minimum sample size for this study was calculated using an 85% study power, confidence level of 95%, with a frequency of periodontitis in the normal weight group and obese group (Papapanou et al., 2018). SPSS 19.0 (SPSS Inc., Chicago, IL, USA) was used for statistical analysis, and the data were expressed as mean ± standard deviation. Normality of the data was checked by Shapiro–Wilk test. Comparisons among groups were performed by one-way analysis of variance (one-way ANOVA) in conjunction with Tukey’s post hoc test (nonskewed variables) and Kruskal–Wallis test (skewed variables) to compare the differences between the treatment groups. *P* values of < 0.05 were considered statistically significant. Logistic regression analysis and Pearson correlation analysis were used for the relationships between serum levels of the interested obesity markers and glycolipid metabolism mediators and the clinical periodontal parameters of the study groups.

## Results

### General data of subjects

There was no significant difference in age and sex distribution, educational level, general health condition covariables, oral health condition covariables, and lifestyle habit covariables among the normal weight group, overweight group, and obesity group (*P* > 0.05). But there were significant differences in the obesity indicators BMI and WHR statistically (Table 1), indicating that the grouping was reasonable and the experimental data had high comparability and significance.

**Table 1:** Demographic of the study groups (n = 112)

Covariate	Control group (n = 36)	Overweight group (n = 38)	Obesity group (n = 38)
Age (years)	41.40 ± 8.42	39.49 ± 5.39	42.50 ± 7.42
Gender (Male/Female)	18/18	21/17	20/18
Schooling level (With/Without university education)	8/28	11/27	10/28
Body mass index (BMI, kg/m^2^)	23.49 ± 0.52	28.53 ± 3.21*	35.80 ± 3.90*#
Waist/hip ratio (WHR, cm/cm)	0.71 ± 0.14	0.81 ± 0.42*	0.85 ± 0.24*#
Hypertension (yes, no)	3/33	5/33	7/31
Pulmonary disease (yes, no)	1/35	1/37	0/38
Cardiovascular disease (yes, no)	2/34	2/36	3/35
Renal disease (yes, no)	0/36	1/37	2/36
Metabolic disease (yes, no)	2/34	1/37	1/37
Immune system disease (yes, no)	0/36	0/38	1/37
Current alcohol habit (yes, no)	10/26	7/31	11/27
Current smoking habit (yes, no)	16/20	11/27	12/26
Physical activity practice (yes, no)	6/30	7/31	6/32
Dental floss usage (More/ Not more than once/day)	2/34	2/36	4/34
Frequency of brushing (More/ Not more than once/day)	31/5	31/7	33/5

All quantitative data are given as mean ± SD.

*Significantly higher than the normal weight group; *P* < 0.05

#Significantly higher than the overweight group; *P* < 0.05

One-way ANOVA and Kruskal–Wallis test were used as the statistically test

### Lipid metabolism in serum

There was no significant difference in blood glucose and HbA1c values among the three groups (*P* > 0.05), indicating that the subjects in the three groups had no clinical manifestations of diabetes such as hyperglycemia or insulin resistance. But there were statistical differences in triglycerides, total cholesterol, and LDL-C measurements among the three groups (*P* < 0.05). HDL-C in the obesity group was significantly lower than that in the overweight and normal weight groups, but there was no significant difference of that between the overweight and normal weight groups (*P* > 0.05) (Table 2).

**Table 2:** Glucose and lipid metabolism obtained in serum of the study groups

Covariate	Control group (n = 36)	Overweight group (n = 38)	Obesity group (n = 38)
Blood glucose (mg/dL)	89.64 ± 3.64	91.61 ± 5.53	94.82 ± 2.14
HbA1c (%)	4.89 ± 0.21	5.19 ± 0.23	5.67 ± 0.17
Total cholesterol (mg/dL)	145.91 ± 23.93	206.51 ± 17.73*	218.86 ± 26.56*^#^
Triglycerides (mg/dL)	105.88 ± 30.02	169.41 ± 50.29*	234.71 ± 51.18*^#^
HDL-C (mg/dL)	68.00 ± 16.44	64.45 ± 10.67	38.22 ± 8.45*^#^
LDL-C (mg/dL)	104.22 ± 10.81	128.92 ± 20.46*	166.37 ± 15.40*^#^

All data are given as mean ± SD.

*Significantly higher than the normal weight group; ^#^Significantly higher than the overweight group; *P* < 0.05

One-way ANOVA and Kruskal–Wallis test were used as the statistically test

### Clinical periodontal measurements

**Fig. 1:**
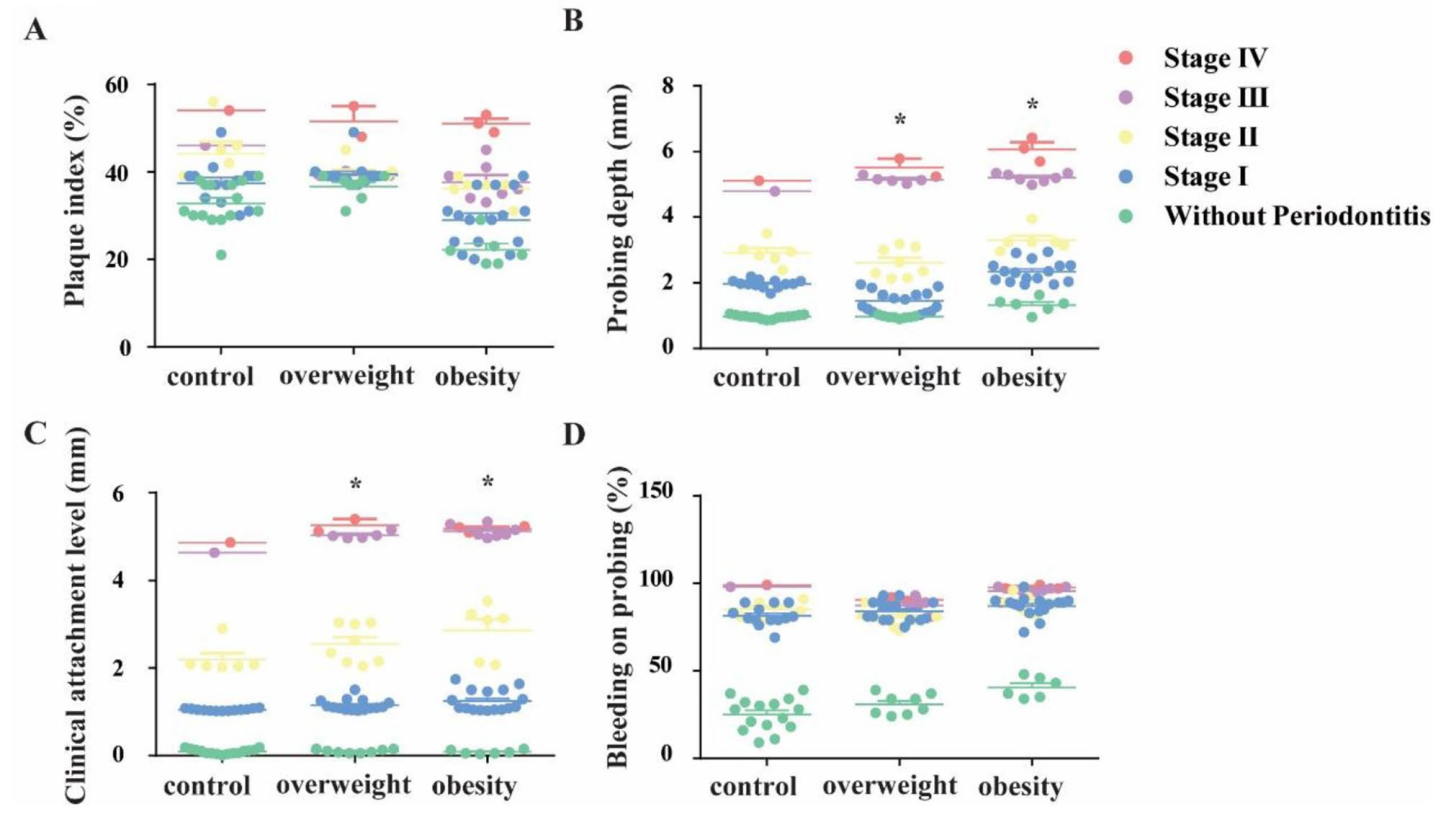
Clinical periodontal characteristics of the study groups. Plaque index (%), Probing depth (mm), Clinical attachment level (mm) and Bleeding on probing (%) of the control group(n = 36), overweight group (n = 38) and obesity group (n = 38) were measured by the professional oral examination. All data were expressed as mean ± standard deviation, * indicated statistically significant difference compared with the control group (*P* < 0.05). One-way ANOVA and Tukey’s post hoc test were used as the statistically test

After the professional clinical periodontal measurements, the participants were classified into different stages of periodontitis. The distribution of the periodontitis classification of the participants among the three groups exhibited an obvious difference according to the results (Table 3). The data showed that the ratio of participants without periodontitis was statistically highest in the normal weight group (41.7%) but least in the obesity group(15.8%). And the proportion of patients with severe periodontitis (stage III and IV) was highest in the obesity group (26.3%) but least in the normal weight group (5.6%). However, when comparing the periodontitis-related clinical indicators of the three groups, it was found that the plaque index of the obesity group and the overweight group showed no statistical difference compared to the normal weight group (*P* > 0.05). While the periodontal pocket depth and clinical attachment level were significantly higher in overweight patients and obese patients than those in normal body weight patients (*P* < 0.05), there was no statistically significant difference in periodontal pocket depth and clinical attachment level between these two groups (*P* > 0.05). Although there was an upward trend in bleeding upon probing among the three groups as the body weight increased (Fig. 1), there was still no statistical difference.

**Table 3:** Distribution of Periodontal Diseases and Conditions of the study groups (n = 112)

Covariate	Control group (n = 36)	Overweight group (n = 38)	Obesity group (n = 38)
Without Periodontitis (n/%)	15/41.7	8/21.1	6/15.8
Stage I (n/%)	13/36.2	15/39.4	16/42.1
Stage II (n/%)	6/16.7	8/21.1	6/15.8
Stage III (n/%)	1/2.8	5/13.2	7/18.4
Stage IV (n/%)	1/2.8	2/5.2	3/7.9

### Concentration of inflammatory cytokines in GCF

**Fig. 2:**
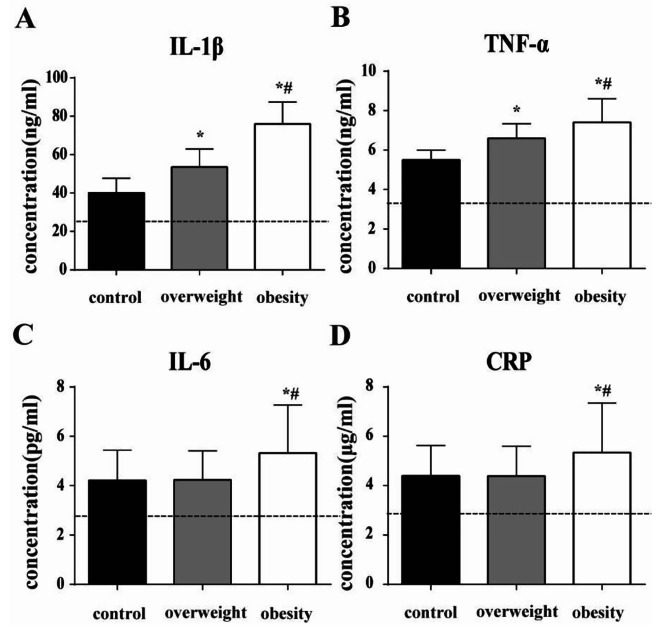
Concentration of inflammatory cytokines obtained in GCF of the study groups. Inflammatory cytokines including IL-1β (**A**), TNF-α (**B**), IL-6 (**C**), and CRP (D) in GCF of the control group(n = 36), overweight group (n = 38) and obesity group (n = 38) were measured by ELISA. The dashed lines in the figure showed the average levels of participants without periodontitis in all the three groups (29 in total) as normal reference. All data were expressed as mean ± standard deviation, * indicated statistically significant difference compared with the normal weight group (*P* < 0.05), # indicated statistically significant difference compared with the overweight group (*P* < 0.05). One-way ANOVA and Kruskal–Wallis test were used as the statistically test

The results showed that the levels of IL-1β and TNF-α in GCF of the subjects showed statistically significant differences among the three groups (*P* < 0.05), while the obesity group was the highest (IL-1β:75.69 ± 11.81ng/ml; TNF-α:7.26 ± 1.90ng/ml), followed by the overweight group (IL-1β:53.69 ± 8.21ng/ml; TNF-α:6.50 ± 1.21ng/ml), and the normal body weight group was the lowest (IL-1β:39.97 ± 6.10ng/ml; TNF-α:5.56 ± 0.98ng/ml). The concentrations of IL-6 and CRP in GCF in the obesity group (IL-6: 5.23 ± 1.91pg/ml; CRP:5.19 ± 1.97 µg/ml) were significantly higher than those in the overweight (IL-6:4.29 ± 1.02pg/ml; CRP:4.34 ± 1.15 µg/ml)and normal weight (IL-6:4.15 ± 1.16pg/ml; CRP:4.29 ± 1.17 µg/ml) groups, with statistically significant differences (*P* < 0.05), but there was no statistically significant difference between the overweight and normal weight groups (*P* > 0.05) (Fig. 2).

### Serum concentration of adipokines and inflammatory cytokines

**Fig. 3:**
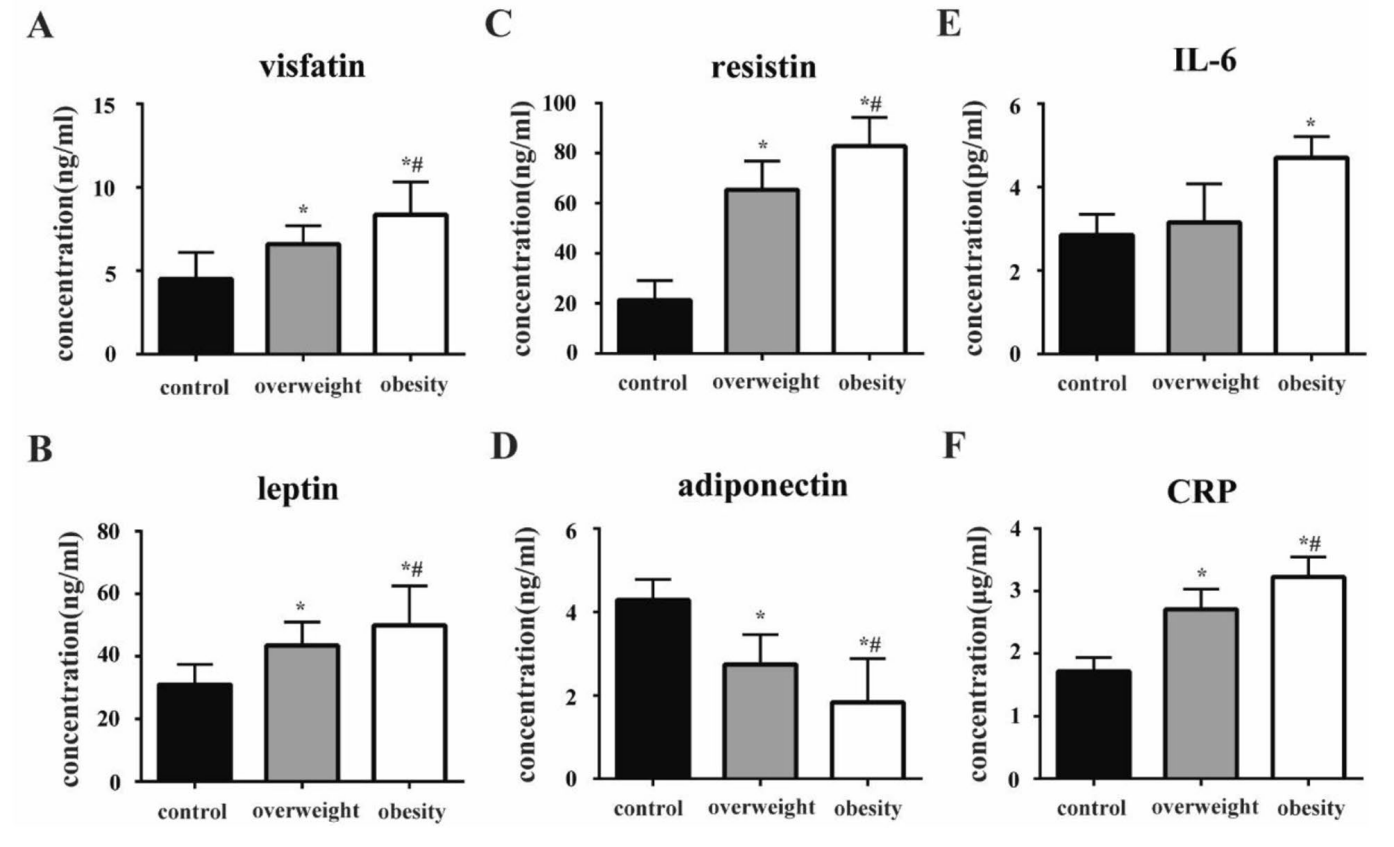
Serum concentration of adipokines and inflammatory cytokines in the study groups. Serum adipokines and inflammatory cytokines including visfatin (**A**), leptin (**B**), resistin (**C**), adiponectin (**D**), IL-6 (**E**) and CRP (**F**) of the control group(n = 36), overweight group (n = 38) and obesity group (n = 38) were measured by ELISA. All data were expressed as mean ± standard deviation, * indicated statistically significant difference compared with the normal weight group (*P* < 0.05), # indicated statistically significant difference compared with the overweight group (*P* < 0.05). One-way ANOVA and Kruskal–Wallis test were used as the statistically test

It was found that serum visfatin, resistin and leptin levels in the obesity group (visfatin: 8.23 ± 1.57ng/ml; resistin: 81.09 ± 5.20ng/ml; leptin: 48.39 ± 3.87ng/ml) were significantly higher than those in the overweight (visfatin: 6.51 ± 0.98ng/ml; resistin: 63.08 ± 5.22ng/ml; leptin: 42.07 ± 1.99ng/ml) and normal body weight group (visfatin: 4.79 ± 1.43ng/ml; resistin: 20.89 ± 3.36ng/ml; leptin: 32.22 ± 1.76ng/ml), while serum adiponectin concentrations (4.30 ± 0.63ng/ml in the normal body weight group; 2.87 ± 0.97ng/ml in the overweight group; and 1.88 ± 1.22ng/ml in the obesity group) showed exactly the opposite changes with statistical significance (*P* < 0.05). At the same time, the serum concentration of IL-6 in obesity group was significantly higher than that in control group and overweight group (2.81 ± 0.39pg/ml in the normal body weight group, 3.19 ± 0.87pg/ml in the overweight group, and 4.47 ± 0.41pg/ml in the obesity group). While the levels of CRP in serum of the subjects showed statistically significant differences among the three groups (*P* < 0.05), while the obesity group was the highest (3.15 ± 0.37 µg/ml), followed by the overweight group (2.67 ± 0.41 µg/ml), and the normal body weight group was the lowest (1.71 ± 0.29 µg/ml), indicating a systemic inflammatory state in obesity group (Fig. 3).

### Correlation between obesity and periodontitis

**Fig. 4:**
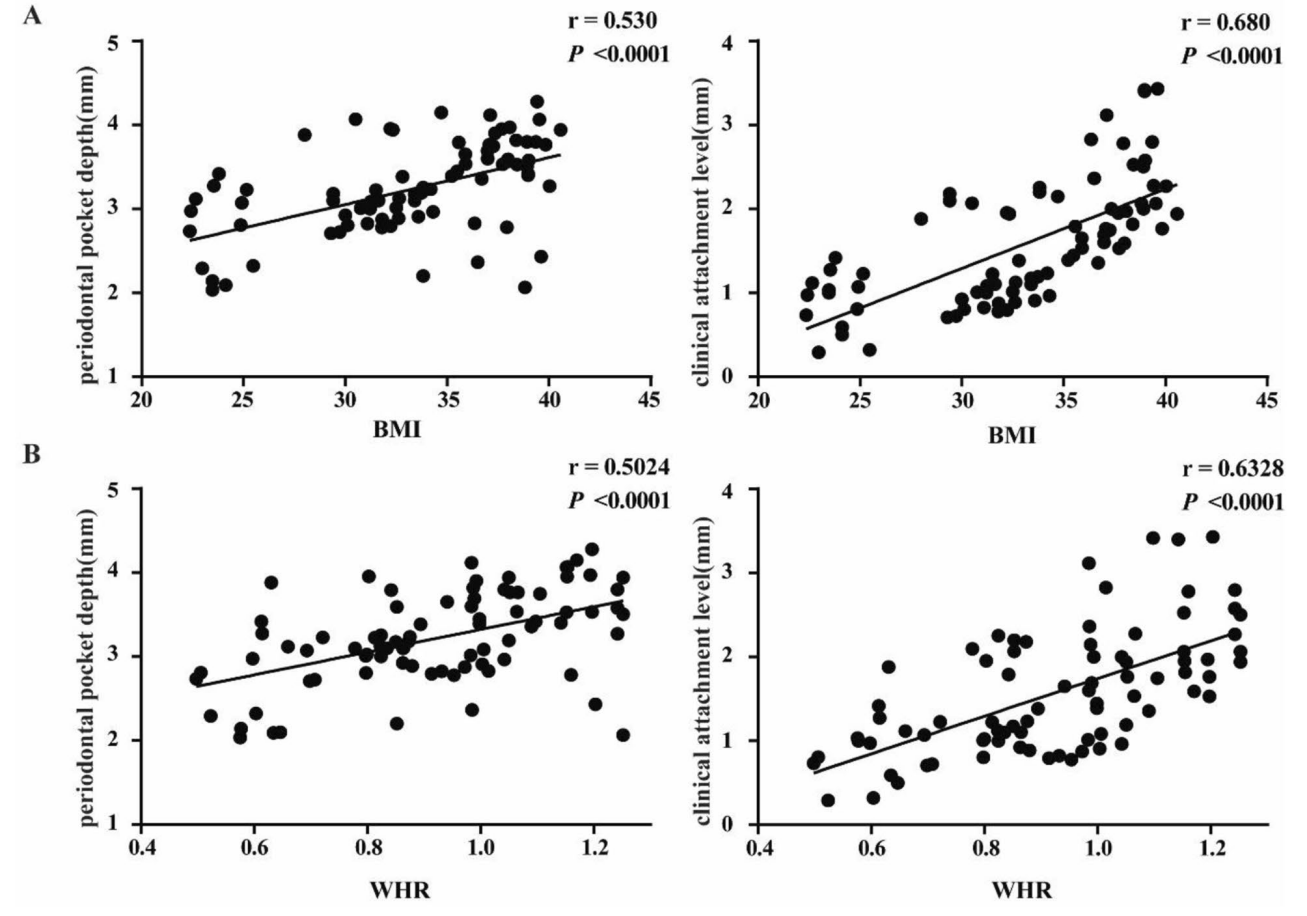
Correlation analysis of BMI and WHR with periodontal pocket depth and clinical attachment level of the study groups. Partial regression plots of periodontal pocket depth and clinical attachment level with the relative levels of BMI (**A**) and WHR (**B**) respectively. All participants from the three groups (normal weight group, overweight group and obesity group) were analyzed for correlation. Correlation analysis results showed that both BMI and WHR were positively correlated with periodontal pocket depth and clinical attachment level (*P* < 0.05, n = 112, Pearson correlation analysis was used as the statistically test)

Through correlation analysis of obesity-related clinical indicators and periodontitis-related clinical indicators in the three groups, it was found that the degree of obesity (both BMI and WHR) of the patients was significantly positively correlated with periodontal pocket depth and clinical attachment level (Fig. 4), suggesting that there is a statistical correlation between the severity of obesity and periodontitis.

### Multivariate logistic regression model of periodontitis

The results of the multivariate logistic regression model showed that periodontitis was associated with significant increases in BMI and WHR, presenting respectively odds ratio of 2.46(1.02–3.89) and 2.78(1.08–4.21). In addition, triglycerides, total cholesterol, LDL-C, visfatin, resistin and leptin were significantly associated with periodontitis, indicating that these glycolipid metabolism mediators and these adipokines are crucial risk factors for the development of periodontitis (Table 4).

**Table 4:** Multivariate logistic regression model for the periodontitis

Variables	β	Std.Error	Odds ratio	95% confidence interval	*P*
BMI	0.902	0.449	2.465	1.585, 3.345	0.002
WHR	1.024	0.480	2.784	1.843, 3.725	<0.001
total cholesterol	0.324	0.146	1.383	1.097, 1.669	0.026
triglycerides	1.139	0.187	3.125	2.758, 3.492	<0.001
HDL-C	0.033	0.309	1.034	0.428, 1.640	0.167
LDL-C	0.592	0.192	1.808	1.432, 2.184	0.007
visfatin	0.664	0.110	1.943	1.727, 2.159	0.006
resistin	0.649	0.308	1.913	1.309, 2.517	0.012
leptin	0.310	0.126	1.364	1.117, 1.611	0.018
adiponectin	0.367	0.853	1.443	-0.229, 3.115	0.294

Bold values denote a statistically significant difference (*P* < 0.05)

Logistic regression analysis was used as the statistically test

## Discussion

Previous studies have shown that obesity and obesity-related systemic diseases increase the risk of periodontitis, and patients with periodontal disease also promote the development of obesity-related diseases, but the mechanism underlining is not clear [12–17, 25]. Gender and age matching of the corresponding number of people was also carried out among the groups to reduce the influence of gender and age on the results of the study. Since diabetes could be associated with obesity and periodontitis, we excluded diabetes as a third factor by comparative analysis of blood glucose and HbA1c in different groups to standardize the study population and to assess the potential impact of obesity, avoiding the interference of the third disease. Most important, to minimize selection bias due to different periodontal statuses in each group, the newest case definitions and diagnostic criteria proposed by the World Workshop on the Classification of Periodontal and Peri⁃implant Diseases and Conditions [24] was employed. Although this criterion has been used in previous studies that associated periodontal disease with other systemic diseases [26–28], it has not been used in this topic yet. The usage of this latest criterion ensures a more accurate and detailed clinical diagnosis of periodontitis. This is very meaningful and important for investigating the relationship between periodontitis and other diseases, which is one of the important innovations of this study.

There was a strong positive association between obesity and periodontitis, after adjustments for covariables and with the specific definition of periodontitis used as shown in the findings above. These results were consistent with recent reports demonstrating that obese rats were more likely to develop periodontitis than non-obese rats. Lundin et al. [29] studied the levels of TNF-α and IL-8 in GCF of 33 participants, and found TNF-α in GCF was positively correlated with their BMI. In addition, Saito et al. [12] and Al-Zahrani et al. [13] also found that there was a significant relationship between obesity and periodontal disease in people aged 18 to 34 years, the mechanism being related to BMI and body fat. In this study, when all individuals were evaluated together, the degree of obesity (both BMI and WHR) of the patients were significantly positively correlated with periodontal pocket depth and clinical attachment level, which further verified the conclusion that obesity is a risk factor for periodontitis in the previous study. After the regression analysis, it was unexpectedly showed that triglyceride was closely related to periodontitis with an OR of 3.125, even higher than BMI and WHR. This was consistent with previous studies, and the mechanism may be related to the association between periodontitis and increased risk of cardiovascular disease [30, 31]. Besides, our findings are also similar to those of Saito et al., who suggested that obesity might be related to the initial stages of periodontitis [32]. For the reasons of this association, Genco et al. [33] proposed a model linking inflammation with obesity and periodontal infections. Proinflammatory cytokines lead to insulin resistance by reducing insulin sensitivity, inhibiting insulin signaling, and enhancing the inflammatory response of the host immune system to dental plaque according to this model. Therefore, the role of adipokine in obesity and periodontitis has attracted more and more researchers’ attention.

Adiponectin can not only inhibit the production of TNF-α and IL-6 but also activate the IL-1 receptor antagonist [34]. Therefore, it has anti-inflammatory, vascular protective, and anti-diabetic effects, which can maintain periodontal health, and the reduction of its receptor can lead to the exacerbation of periodontitis [35, 36]. Leptin is one of the first adipokines to be discovered. In addition to regulating food intake, energy expenditure, lipid metabolism, and bone metabolism, it can also regulate immune inflammatory processes (mainly pro-inflammatory effects). Studies have found that leptin can be expressed in both normal and inflammatory gingival tissues, but the expression level is decreased in inflammatory gingival tissues, while the serum expression level is increased [37]. The imbalance between proinflammatory and anti-inflammatory adipokines in this study `also supports the comorbidities effect of periodontitis and obesity. The levels of serum visfatin, resistin and leptin in the obesity group were higher than those in other groups. In contrast, the anti-inflammatory adipokine adiponectin had lower blood concentrations. The gingival epithelium is the first barrier against bacteria, and adiponectin also has a certain effect on the content of bacteria in gingival epithelial cells. Bacterial infection can stimulate to secretion of inflammatory mediators and proteases, and gingival epithelial cells can remove them by innate and acquired immune responses [38]. In addition, some scholars have also found that leptin and resistin have a certain impact on the pathogenesis of periodontitis [39, 40]. What’s more, one of the biological explanations for obesity negatively affects the progression of periodontitis and the response to periodontal therapy may be also due to the unaffected leptin, resistin and adiponectin levels after the periodontal non-surgical treatment [41]. In addition, enamel matrix protein in periodontal ligament cells can promote periodontal tissue regeneration, while visfatin can inhibit the activity of enamel matrix protein. Elevated visfatin levels in obese patients with periodontitis may adversely affect the regeneration of periodontal tissue [41]. Therefore, we speculate that a high concentration of leptin and visfatin in obese patients may increase the risk of periodontal disease. Besides, previous literatures also reported the possible role of other factors in the pathogenesis of obesity and periodontitis, such as melatonin. Obese rats with periodontitis demonstrated significantly lower melatonin concentrations when compared with either periodontitis or obese rats. While adjunctive melatonin therapy significantly reduced alveolar bone loss and pro-inflammatory cytokines mainly in animals with both periodontitis and obesity. However, the mechanism of melatonin, as well as the possible internal linkage between melatonin and other adipokines involved in periodontitis and obesity are still unclear, which are expected to be further clarified [42, 43]. Although there are some discoveries in this research, this is a rather small-scale study with a relatively low sample size(36 normal weight patients vs. 38 overweight patients vs. 38 obese patients), which is a remarkable limitation in this study and may inevitably influence the reliability of our results. Further studies are in need to be verified using a sufficiently large sample size, and to uncover the molecular mechanism of adipokines in the correlation between obesity and periodontitis.

## Conclusions

In conclusion, obese patients have worse periodontal conditions and significantly high levels of inflammatory cytokines and adipokines expression. What’s more, the severity of obesity is positively correlated with the severity of periodontitis. This study suggests the possible aggravating outcome of periodontitis in the systemic inflammatory response of obese patients, and the overexpression of adipokines may be an important factor in the continuous deterioration of obesity and periodontitis.

## Data Availability

All data included in this study are available upon request by contact with the corresponding author.
